# “When the Emerald wood dove calls, the earth prepares to drink”: Community elders lived experiences of indigenous weather forecasting in eastern Mount Kenya region

**DOI:** 10.1371/journal.pone.0355610

**Published:** 2026-09-08

**Authors:** Beatrice N. Warue, Stephen K. Mucheke, Richard Muita, Adelaide M. Lusambili

**Affiliations:** 1 Innovation and Community Engagement, Africa International University, Nairobi, Kenya; 2 Innovation and Community Engagement, Africa International University, Nairobi, Kenya; 3 Kenya Meteorological Department, Ministry of Environment, Climate Change and Forestry, Nairobi, Kenya; 4 Knowledge Translation & Policy Unit; Nextgen for Earth, Nairobi, Kenya; University of Florida, UNITED STATES OF AMERICA

## Abstract

**Background:**

Indigenous weather forecasting plays a critical role in supporting climate-sensitive livelihoods in Eastern Mount Kenya, yet there is limited empirical understanding of the methods, perceived accuracy, and social mechanisms sustaining their use. This study examines these practices and their contribution to community resilience under increasing climate variability.

**Methods:**

We conducted 81 in-depth interviews with elders aged 65 years and above across the region. Thematic analysis was applied to coded data to identify patterns, indicator types, and perceptions of reliability and resilience, while comparing insights across communities.

**Results:**

The study makes three original contributions to ethno-meteorological knowledge which include atmospheric, biological and celestial indicators. In addition, it identifies somatic forecasting as a dominant and systematic knowledge domain, conceptualizing the human body as an environmental sensor within indigenous climate knowledge systems. It also demonstrates that declining reliability of indigenous indicators arises from ecological disruptions, highlighting the vulnerability of indigenous knowledge systems to environmental change. Further, the findings reveal a scale mismatch between scientific forecasts and community-level decision needs, showing that hybrid approaches integrating indigenous and scientific knowledge are more trusted and actionable at the local level. Additional insights include the role of elders as informal climate governance actors and the emergence of intergenerational knowledge loss as a key dimension of climate vulnerability.

**Conclusion:**

By conceptualizing indigenous weather forecasting as a dynamic, socially embedded, and ecologically grounded system, this study provides empirical evidence that can inform the development of a framework for locally responsive hybrid climate approaches. The results indicate the value of systematic documentation and cultural preservation and point to the potential benefits of integrating such knowledge into policy and climate-service design to enhance adaptive capacity and sustain community-based climate resilience.

## Introduction

The negative impact of climate change manifesting in frequent extreme weather events, higher temperatures and unusual rainfall patterns, is being experienced globally. These effects have far-reaching consequences tin many developing countries. They put a strain on natural resources and slow the achievement of national development goals [1,2]. Such negative consequences specifically affect national food security, education, health and other sectors of development. They also interfere with agriculture systems in the developed world and disrupts global food chains through which support for emergencies in the developing countries is channeled [3,4]. There is therefore an urgent need for concerted efforts between global organizations, regional bodies and national governments to develop coping strategies to prevent disruption of social economic goals and alleviate human suffering in the wake of climate change [5–7].

In Africa, the impact of climate change is felt more in rural communities whose livelihood depend on rain-fed agriculture. This impact is more severe in low-income populations and other vulnerable communities [8]. The African Development Bank (ADB) reports that extreme weather events such as prolonged droughts, destructive floods, and shifting rainfall patterns are causing annual economic losses totaling to a significant percentage of GDP in many African countries [9]. This negative effect is made worse by structural vulnerabilities including poverty, weak infrastructure, and governance challenges. Without adaptation and resilience-building measures, climate change threatens to reverse decades of development gains and increase in existing inequalities across the continent [10,11].

Over the past decade, countries across Eastern and Southern Africa have experienced intensifying climate variability. The outcome of these changes is degradation of soils, poor harvests, water scarcity as well as destruction of range land and forests and the entire natural-resource base. Rain-fed agriculture especially small-scale farming for domestic food production suffers a more severe impact resulting in hunger and threat on livelihoods. The higher temperatures and inconsistent rainfall also affect income generating cash crops such as tea coffee and wheat thereby destroying the resource base of affected communities. [12,13].

In the livestock dependent areas, pastoralism and mixed livestock activities are affected by recurrent droughts, poor pastures and frequent occurrence of diseases resulting in lower productivity and major losses. On the other hand, the warming of in-land water bodies including lakes Victoria, Tanganyika and Malawi is affecting fish reproduction resulting in lower catch leading to shortages that culminate into income loss and dwindling quality of diet among fishing communities. Furthermore, forest degradation, through charcoal burning and lumbering, and unreliable rains are causing shrinking of arable and natural habitat creating a potential for human-wildlife conflict [14,15]. Recent reports indicate that in many parts of Kenya, Ethiopia, and Tanzania, erratic rainfall patterns are disrupting traditional cropping calendars, leading to mismatches between sowing periods and optimal growing conditions [16,17]. With these challenges, the Food and Agriculture Organization (FAO) warns that these climate-induced disruptions not only threaten immediate food availability but also undermine long-term agricultural sustainability. The total effect of these disruptions may entrench poverty further and increase the socio-economic inequalities in the region [18].

The increasing weather variability also interferes with the reliability of meteorological forecasting and long-established seasonal weather patterns, resulting in uncertainty of short-term forecasts and seasonal projections that guide communities. Recent assessments of this phenomenon in Africa shows that altered onset and cessation of rains, coupled with greater frequency of extreme events undermine the accuracy of meteorological models [19]. This weather variability is also affecting indigenous weather forecasting methods where the established patterns and no longer align with expected outcomes. The disruptions on modern scientific and indigenous forecasting methods weaken the early warning systems. These disruptions affect decision-making in agriculture, pastoralism, and water management. It also contributes to vulnerability for millions of households in the region. The declining reliability of modern meteorological forecasts increased attention on indigenous weather which are rooted in generations of observations and interpretation of environmental indicators. It is a science based on experiences and interaction with the environment. It incorporates signals, patterns of behavior and changes within the atmosphere, on plants, animals, insects, and celestial bodies which herald change in seasons [20,21].

Combining indigenous and scientific weather methods is becoming a frequent topic for climate changes mitigation strategies due to increasing evidence on its efficacy. In Zimbabwe, maize famers reported to have achieved a higher average consumer surplus gain after applying indigenous methods to determine exact planting time [22]. This development highlights the practical importance of integrating indigenous weather forecasting into formal climate services. It also provides some evidence of underutilized potential and opportunities for co-produced hybrid forecasting to improve community-level climate preparedness, suggesting a need for such localized, research-based documentation to safeguard vital indigenous knowledge from being lost as elder custodians pass away [23].

Other studies across Eastern Africa have shown that smallholder farmers and pastoralists often turn to indigenous methods when official forecasts are not available or are perceived as too generalized to guide local decision-making. In comparison, the indigenous systems offer location-specific insights and are considered relevant under changing climatic conditions. Despite their being more preferred, their predictive accuracy is also challenged by unprecedented climate variability witnessed globally [24,25]. Modern weather monitoring systems provide predications based on science and real-time technological data guided models. On the other hand, indigenous knowledge draws from generations of localized environmental observation and interpretation. There is increasing evidence on the value of integrating these indigenous forecasting approaches to improve forecast accuracy and strengthen community adaptation strategies in the wake of destabilized weather patterns caused by climate change [26].

In this regard, the Kenya Meteorological Department and Baringo County leadership have established a working partnership through Participatory Scenario Planning (PSP). By incorporating indigenous weather indicators into PSP exercises, the initiative has fostered co-production of climate information and strengthened the relevance of forecasts for local decision-making. This model demonstrates how formal meteorological services can collaborate with local leadership to translate indigenous knowledge into actionable inputs for community planning and adaptation.

This co-production of climate information has enhanced both forecast accuracy and user confidence, enabling more informed agricultural and pastoral planning [27]. Such hybrid approaches are increasingly recognized as strategies to strengthen climate resilience in rural Africa. The empirical evidence of indigenous forecasting is strengthened by research among the Gujii pastoralists of southern Ethiopia, where indigenous forecasts based on livestock entrails, insect and animal behaviors, and celestial cues were successfully compared with formal meteorological data. The outcome revealed points of convergence between modern and indigenous methods. It also revealed areas of decline of indigenous methods due to external influences such as technology. [28,29]. This evidence highlights the need to integrate indigenous weather forecasting and meteorology services. It also further suggests a need for, research-based documentation of indigenous weather methods to authenticate and safeguard the knowledge from falling through the intergenerational gaps.

Indigenous weather forecasting methods continue to play an important role in strengthening community resilience [30]. Although their perceived accuracy is sometimes questioned under current climate variability, these methods remain valued because they are highly localized and specific to the cultures. Knowledge transmission is usually conducted by elders and serves important function of community preparedness. When combined with modern forecasts, indigenous methods enhance adaptive capacity by providing locally relevant guidance that strengthens preparedness and supports sustainable resource management [31,32].

A closer review of the literature shows that much research on indigenous weather knowledge has relied predominantly on survey-based methods. Such approaches often fail to capture the rich cultural context, the depth of local knowledge, and the ways this knowledge is integrated into everyday life in the face of climate change. Further qualitative research is therefore needed to explore community lived experiences, coping strategies, and emergent forms of community resilience to climate change [33].

Although considerable research on weather forecasting has been conducted across multiple regions of Kenya [34,35], Eastern Kenya remains comparatively underrepresented especially with respect to studies of indigenous forecasting practices. Addressing this geographic and methodological gap is essential for generating context-specific evidence that advances scholarly understanding and informs locally appropriate adaptation strategies and policy interventions. [36]

This study addresses the gap in qualitative research noted above by focusing on the eastern Mount Kenya region. It makes a valuable addition to climate change coping strategies by documenting indigenous weather forecasting methods, particularly the rarely recorded, elder-held knowledge that continue to inform local climate knowledge. It also offers valuable insights on the perceived accuracy and reliability of such methods, motivations for continued use and their contribution to community resilience in the wake of erratic weather patterns and impacts of climate change.

The objectives of this research were: (1) to document the indigenous methods used to predict the weather change; (2), to assess community perceptions of their accuracy and reliability; (3) to examine the factors influencing continued of such methods; and (4) to examine how this knowledge contributes to community resilience. The study addressed the following questions: (1) what indigenous weather forecasting methods are currently practiced by rural communities in the selected counties of the Eastern Mount Kenya region? (2) how do community members perceive the accuracy and reliability of indigenous weather forecasts compared to modern meteorological forecasts under current climate variability? (3) what factors influence the continued use, adaptation, or decline of indigenous weather forecasting methods in the context of changing climate conditions? and (4) application of this knowledge applied in building community resilience. A deeper understanding of these questions would contribute valuable insights to support climate change coping strategies within communities.

## Methods

### Approach to field research and conceptual framework

The study protocol for this research was approved by the National Commission for Science, Technology & Innovation (NACOSTI), under License No: NACOSTI/P/24/37767 and Day Star University Ethics Committee Ref. DU-ISERC/03/07/2024/00111. Endorsement for the study was sought and granted by the county commissioners (Embu County ref EBU.CC/ADM/3/37/VOL.1V/(56). In addition, community elders in all research areas were also contacted for their approval. The study employed qualitative design using key informants to solicit detailed information from community members on their experiences and perspectives on indigenous weather forecasting approaches. The research started with a mapping of the study area and recruitment of participants followed by data collection after all approvals by relevant authorities. Data collection was conducted from 5^th^ August to 30^th^ of September 2024. We used Consolidated Criteria for Reporting Qualitative Research (COREQ): a 32-item checklist for conducting qualitative research [17] (S1 Appendix)

**Fig 1:**
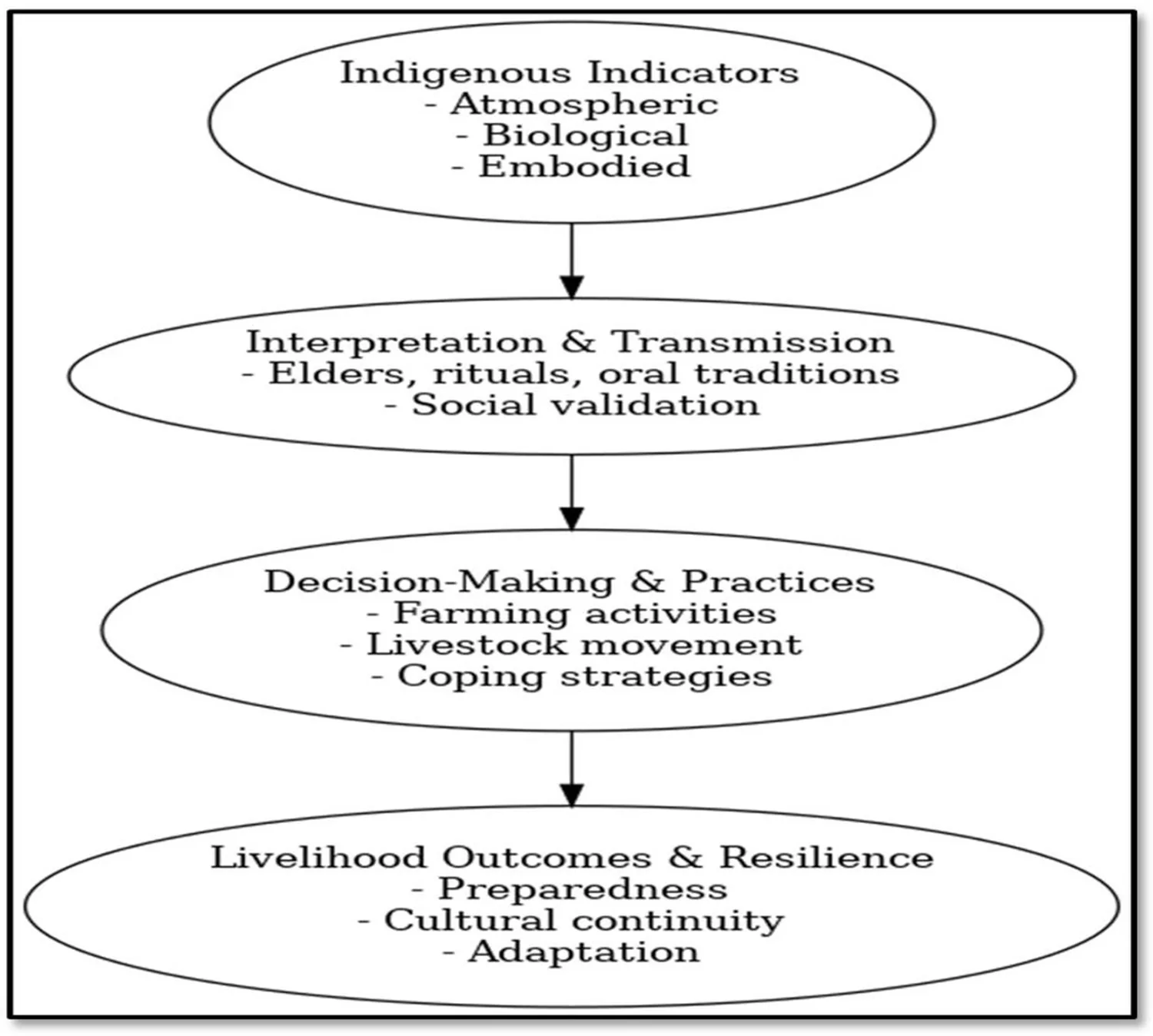
Conceptual framework of indigenous weather forecasting.

We adapted a conceptual framework on indigenous methods of weather forecasting [37,38], to guide the research. The framework presents indigenous indicators, which include biological signs such as animal behavior and plant flowering, astronomical cues like star and moon patterns, atmospheric observations of clouds, winds, and humidity. The framework also incorporates scientific forecasts by meteorological services as a means of verification. It illustrates the flow from observed indicators through decision-making and livelihood activities, to outcomes. These outcomes contribute to community resilience especially the capacity to adapt, sustain livelihoods, and transmit knowledge across generations. A feedback loop of validation and integration connects indigenous methods with community deliberation and scientific forecasts, enhancing trust and adaptive strategies. This representation (Fig 1) puts emphasizes on the relationship between local knowledge systems and broader climate information in shaping resilience [39–41]

### The study population

The study was conducted in the Eastern Mount Kenya region, encompassing communities in Meru Embu, Mbeere, and Tharaka. As shown in Table 1, Meru County has a total population of 987,653 and 13 sub-counties with 53,500 males and females aged 65–79 years [42]. On the other hand, Embu County has a total population of 608,599 and 6 sub-counties with 47,723 males and females aged 65–79 years. Tharaka region has a population of 393,177 and 6 sub-counties with 16,586 males and females aged 65–79 years [18]. Mbeere has a population of 18,211, 9945 females and 8266 males between 65 and 79 years [42].

**Table 1:** The study area population.

Regions	Age Cohort	Male	Female	Total
Embu	65-79	11,077	18,435	29,512
Mbeere	65-79	8,266	9,945	18,211
Meru	65-79	25,388	28,112	53,500
Tharaka	65-79	7759	8827	16,586
	**Totals**	**52,490**	**65,319**	**117,808**

**Note:** Mbeere is within Embu County

**Source:** Meru, Tharaka Embu and Mbeere Counties Integrated Development Plan 2018–2022

### Study sites

**Fig 2:**
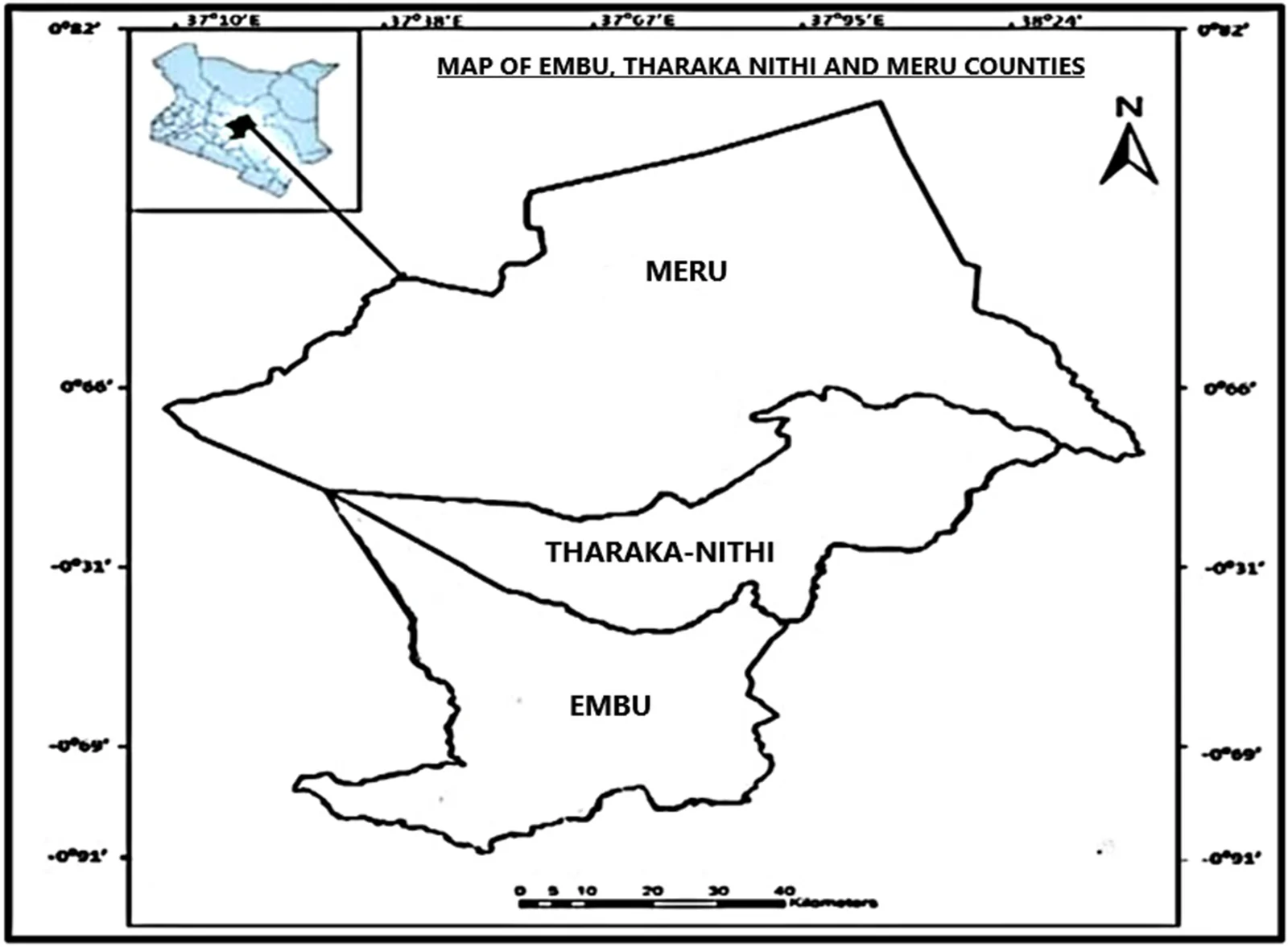
Map of Mount Kenya East Region.

The Eastern Mount Kenya region experiences diverse climatic conditions shaped by altitude and its proximity to Mount Kenya (15,098 ft above sea level). Tea in Embu and Meru counties is primarily grown on the highland slopes of Mount Kenya, at elevations between 1,500 m and 2,100 m. Runyenjes in Embu and the upper zones of Meru fall within this optimal altitude range. The higher-altitude areas of Embu and Meru enjoy a cooler, wetter climate, with annual rainfall ranging from 1,200 mm to 2,200 mm and mean annual temperatures between 14 °C and 20 °C, conditions favorable for crops such as tea, coffee, and vegetables. In contrast, the lower-altitude regions, including Mbeere and Tharaka, are semi-arid with lower and less reliable rainfall, typically ranging from 600 mm to 900 mm annually. Mean annual temperatures in these lowland areas range from 24 °C to 32 °C, creating conditions more suitable for drought-resistant crops such as millet and sorghum. Agricultural activities and water resource management are strongly influenced by the long rains (March–May) and the short rains (October–December). The short rains support farming in the highlands, while the lowlands focus on harvesting drought-tolerant crops during drier periods. In the lower parts of the region, such as Tharaka and Mbeere, reliance on seasonal rivers and water harvesting techniques has increased, while recurrent droughts have led to water scarcity and, at times, competition and conflicts over resources. The communities of Meru, Tharaka, Embu, and Mbeere share a common ancestry and are closely related through their language, cultural practices, and geographical proximity (Fig 2). These groups, collectively referred to as part of the larger Eastern Bantu cluster, exhibit strong socio-cultural and linguistic similarities that trace back to a shared historical origin. They were selected for research on indigenous weather forecasting methods, as their traditional knowledge systems are expected to reflect common patterns and shared environmental interactions. [43].

### Recruitment of Participants

We recruited and interviewed a total of 81 key informants, 65 years and above, across the region, comprising 25 men and 12 women in Meru and Tharaka and 26 men and 18 women in Embu and Mbeere. We used Community Health Workers (CHWs) in Embu and Mbeere and snowball sampling facilitated by community elders in Meru to recruit the study participants. CHWs play a vital role at the grassroots level. They are trusted by local communities which made them valuable partners in the recruitment process. In Meru the CHWs were not immediately available to support the research. We used community informants identified by a village elders, then adapted a snowball method sampling to reach other participants. This approach relied on peer referrals and word-of-mouth recruitment, which proved especially suitable in the close-knit social structures typical of the region. We noted that older adults were more receptive to participating when referred by someone within their social network, facilitating trust and willingness to engage. Snowball sampling also allowed access to older adults who may not be part of formal health or community systems.

Using CHWs and a snowballing methodology proved efficient and effective in that they are trusted members of the community with established relationships and cultural familiarity. This helped create trust between the researchers and elderly key informants. Engaging elders aged 65 years and above through in-depth interviews provides access to authoritative, time-tested interpretations. This segment of the population has a wealth of lived experiences and cultural values. Their depth of understanding and ability to translate environmental signals into knowledge to guide decisions makes elders valuable contributors to community resilience. In-depth interviews proved the most effective research method for this study, as they allowed for rich, nuanced insights into complex indigenous practices [44].

Individuals were included in the study if they were born and grew up in the study area to allow them to be able to share their lived experiences. Occupation, literacy levels, and gender were not an exclusion criterion however, those who were below 65 years of age and were born in the region but lived outside the area since the age of over 20 years were excluded as they were deemed not to have the native experience.

Participants were selected based on an inclusion and exclusion criteria and were screened by interviewers to ascertain their suitability. We provided all potential participants with a clear explanation of the study’s purpose and objectives during recruitment and again before data collection began. Information from the consent form (S2 Appendix) was read aloud in the participants’ mother tongue, and time was given for questions and clarifications. Consent was obtained through either a signature or thumbprint, depending on literacy levels.

Throughout the interviews, the researchers regularly confirmed participants’ ability and willingness to continue with the interviews. At the end of each session, participants were requested to confirm that their responses could be used for the study. In total, 69 of the 81 participants gave the final approval of the information to be used in the research on condition that all knowledge shared is attributed to the community.

### Data collection

#### Piloting of research tools.

We piloted the research instrument with selected individuals to test their suitability. We learnt that the term “climate change” was not used in the like it is in academic and research circles. Instead, respondents were more familiar with the notion of changing weather patterns, particularly in relation to the disruption of long-established land-use practices. We aligned our instrument with participants’ conceptual understanding while maintaining relevance to the broader discourse on climate change. The framing enabled participants to articulate disruptive weather patterns and consequent impacts on livelihood activities over time. We were also proved our choice on interview method in that In-depth interviews were appropriate for documenting indigenous weather forecasting among elders aged 65 years. It allowed them to narrate detailed accounts of lived experiences in weather forecasting and cultural meanings attached to the skills and observed changes.

The Information Sheet (S3 Appendix) was used before data collection. The researchers (BW, SM), proficient in the local dialects, conducted 81 interviews in both Ki-Meru and Ki-Embu language. Twelve individuals spoke both local and English language and during their interviews, they were informed by researchers to speak in the languages they felt comfortable SM and BW conducted interviews as part of the research process. SM, an experienced public health specialist with wide experience in qualitative research, worked alongside BW, who has undergone advanced training in the conduct and implementation of qualitative studies.

All interviews were held at participants’ homes, mostly within compounds and few inside the house. For some participants, particularly among the elderly couples, their spouses were also present during the interview process, and it was not possible to separate them. Each interview lasted for at least two hours and no more than three hours, with pauses taken whenever participants requested. In the high-altitude areas of Embu and Meru, which experienced chilly morning weather, interviews were conducted in the mid-morning when it was warmer. In lower-altitude regions of Tharaka and Mbeere, which experienced higher temperatures late afternoon was preferred due to the cooler weather conditions. All interviews were conducted in Kimeru and Kiembu language.

Interviews were conducted in Kimeru and Kiembu and translated verbatim into English by SM and BW, who are native speakers of these languages. For each question, the interviewer explained the context and ensured standard translation using a consistent choice of words to maintain the original meaning. Interviews conducted in the local Meru and Embu dialects were transcribed word-for-word into English by B.N.W and S.K.M. The researchers shared the transcripts amongst themselves and compared them to ensure the original meaning was accurately preserved during transcription. This approach ensured that all participants were asked the same questions within the same context in the local language

### Data analysis

We conducted a qualitative thematic analysis of the data using a constant comparative method to make the analysis systematic, iterative, and credible [45]. We began the analysis with data preparation and organized it systematic file structure. Before actual coding, we read each transcript multiple times to capture first impressions and recurring phrases. Data were examined line by line, and provisional codes were assigned to capture both explicit expressions and underlying meanings. This was followed by initial coding. We developed a codebook including definitions and illustrative quotes. The related codes were clustered into broader categories. We merged some of the codes and refined others in the process of the analysis. Two authors B W and S M reviewed the complete code book and sections coded in-depth interviews together. We employed triangulation of data sources and member checking, to ascertain credibility and trustworthiness of findings. Finally, we engaged in interpretation and validation, linking the emerging themes to cultural practices and resilience strategies by the communities.

### Study findings

The study revealed that indigenous weather forecasting remains an integral and actively practiced tradition among rural communities in the Eastern Mount Kenya region. Communities depend on a wide range of indicators including atmospheric observations, celestial patterns, biological and somatic cues that align closely with the region’s seasonal calendar. The respondents reported there is a continued reliance on these methods, noting that they provide localized, context-specific insights that are often absent from conventional meteorological forecasts, particularly under the increasing unpredictability brought by climate variability.

**Fig 3:**
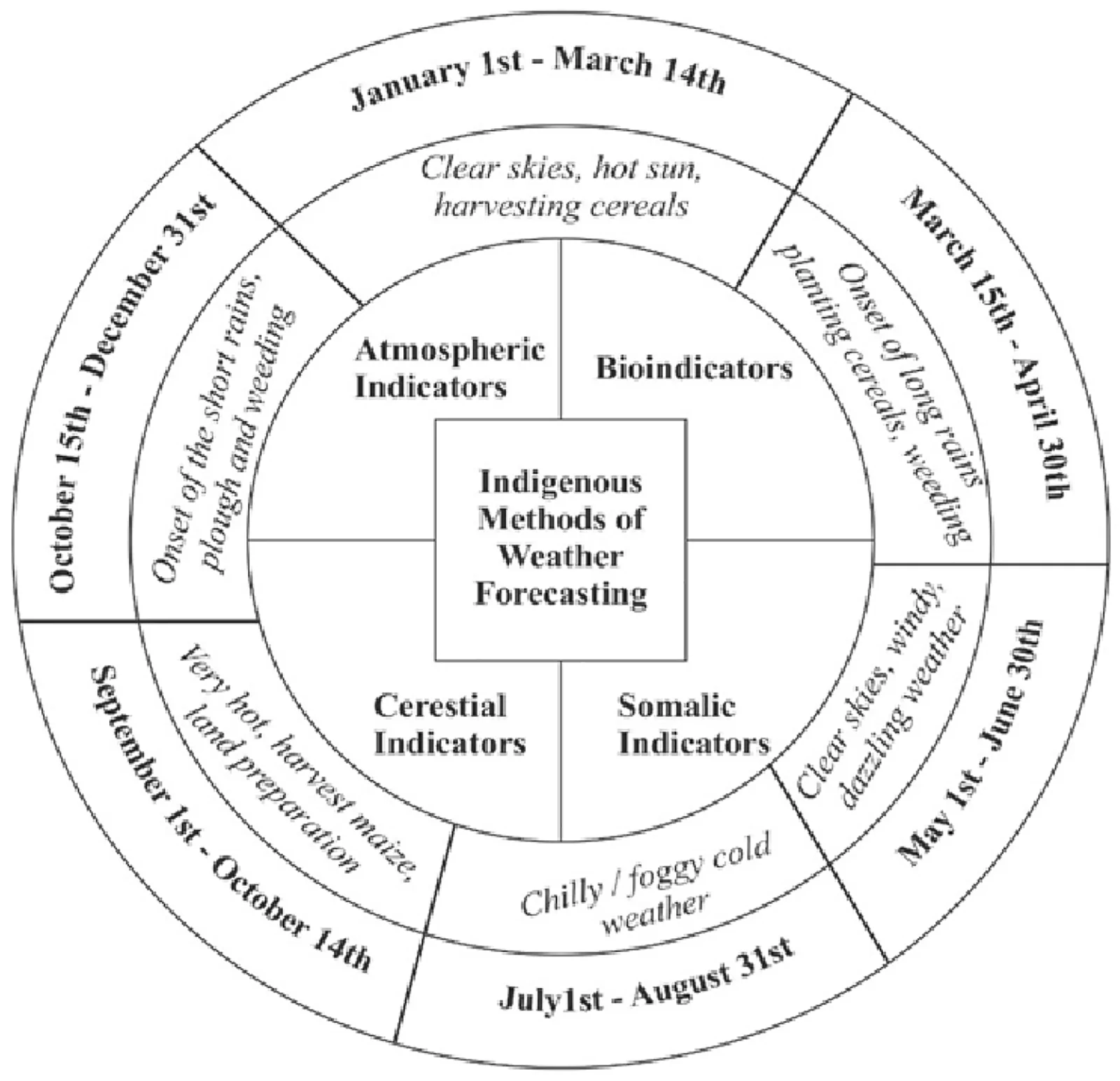
A graphic illustration of indigenous weather indicators and seasons.

While respondents acknowledged that both indigenous signs and modern forecasts have lost some precision due to shifting weather patterns, they emphasized that combining the two sources enhances overall accuracy and strengthens decision-making for livelihood activities such as crop selection and timing, livestock mobility, and early preparation for extreme weather events. The findings further show that the persistence, adaptation, or decline of indigenous forecasting is shaped by multiple factors, including intergenerational knowledge gaps, reduced transmission of skills to youth, environmental changes affecting the visibility of natural indicators (Fig 3). Despite these pressures, communities continue to value indigenous methods as culturally rooted, practical, and complementary tools for navigating an increasingly variable climate as illustrated

### Indigenous weather methods and application

The following sections present a detailed analysis of the study’s key findings, organized around the major categories and themes that emerged from the data. These themes highlight how communities in the Eastern Mount Kenya region understand, apply, and evaluate indigenous weather forecasting knowledge within the context of a changing climate

(i) **Biological weather indicators (birds, insects, animals and plants)**

Birds were a common weather change indicator across the entire region. Migratory birds like swallows which start appearing in start appearing from mid-September, usually migrating from north to south in increasing numbers, particularly noticeable in the late afternoon, signaling the imminent arrival of the October rains. These migrations are a reminder to farmers to start preparing their land for the planting season starting from mid-October. Apart from land preparation there are other activities like spraying of coffee, usually done before the rains to aid flowering.

*“When you hear the Swallow bird (Muguru) calls while hurrying south, know it’s time to spray your coffee.*

Among the birds, the Emerald Dove (Gichea/Kijea) holds a special place in the communities. Its soft, gentle cooing after long periods of tormenting heat is received with a degree of emotion by the community. It symbolizes end of dry season hardships and the coming of rains.

“ *When the Emerald Wood Dove calls, the earth prepares to drink….to quench the thirst!*

Although some birds are associated more with good news and others not. The Grey Hornbill cries in distress, indicating poor rainfall and impending drought. The hornbill’s distinctive calls are thought to reflect environmental stress, and their behavior can serve as a signal to local communities about changing weather patterns.

Dragonflies are a reliable indicator of the onset of the rainy season. Similarly, the emergence of termites from their mounds after dry spells was consistently mentioned of rain. The chirping of crickets at night, the buzzing of certain beetles, and the intensified activity of ants carrying food to higher grounds are signals to change in seasons.

**‘** *When dragonflies dance and dive with their tails touching the ground, it means there will be a heavy downpour within a week”*

Although flying insects are widely observed as indicators of seasonal change, their signals are interpreted with both positive and negative meanings.

*“Butterflies appearing after the rains when crops are green reminds farmers to spray their crops because their appearance is associated with crop destroying insects.*

Thus, these insects embody both hope and caution within indigenous forecasting knowledge. Participants reported that domestic animals especially cows and goats display heightened excitement prior to the onset of rains.

*“They sniff the air with heads raised toward the wind; a behavior interpreted as sensitivity to changing atmospheric conditions*”. They also reported other animal behaviors as,

*“if you see the cows running with tails raised and mooing with excitement, prepare your seeds for planting”*

Participants reported that herders and their animals respond in tandem to shifting weather: local proverbs encourage herders to attend to signs of animal excitement as indicators of imminent rain, reflecting an embodied, reciprocal knowledge of environmental change. These observations suggest that beyond their role in providing food and livelihood resources, domestic animals contribute to indigenous weather prediction systems, reinforcing their broader significance within community adaptation strategies.

Trees play a significant role in providing signals for changing weather. In Meru and Embu, species such as Croton (Mutuntu), Meru Oak (Muuru), Sycamore (Mukuu), and Coral Tree Erythrina Abyssinia (Muvuti) provide reliable signals of rainfall through their cycles of flowering, leaf shedding, and renewal. The striking red blooms of the Erythrina tree is a sign of coming rains. These signals prompt farmers to prepare land ready for planting. “*When the flame tree blooms, the rains will soon follow.*

*“The beautiful red flowers on coral tree is a sure sign of rains coming.*

In the drier parts of the region including Mbeere and Tharaka, the flowering of yellow Bark Acacia (Mureera) tree is a sure sign is a reminder of the need to prepare the land for prompt planting.

“ *When the acacia whitens with bloom, the soil calls for the hoe.”*

At another level, the release of pollen from trees signals seasonal change. Flowering trees mean rains are near; *when pollen causes sneezing, people take it as another sign the season is changing.”*

*He who sneezes at acacia bloom should smile, for the soil is drinking.” “The nose may itch, but the rains will feed the belly.”*

**(ii) Atmospheric weather indicators**

Cloud patterns, wind intensity and direction; temperature and humidity, lightning and thunder, the color of the sky were all indicated to be live indicators of weather change. They opined that the intensity of such signals is more pronounced at the end of the dry season when the rains are near. Additionally, the formation, color, and height of clouds are observed closely, with darker clouds and the growing height of cloud formations being indicative of impending rain. Thunder and lightning are also significant, with communities often noting these atmospheric disturbances as signals that the rainy season is near. Together, these indicators form a comprehensive understanding of weather patterns, guiding local communities in preparing for agricultural and livelihood activities.

*“There dark clouds over Nkuriga hill, the Kiamiogo community will be well nourished. “…low lying thick Clouds and lying on hill seen in Kianjiru and Kiangombe hills indicates the rains coming.*

*“The rain has extended its nourishing hand … depicting a human palm….; no person who has planted will sleep hungry again.*

It is worth noting the mix of physical features such as hills and the personification of rain as extending a nourishing palm reflects a sense of unity of purpose between the landscape and atmospheric conditions in bringing good news to the community. The statement is provided with a sense of finality and authority which reflects on the importance of those who have this kind of skills to speak with authority.

We learned that the direction from which the wind blows, and its strength are closely observed by communities as reliable indicators of changing weather. Highland areas in Embu and Meru experience cool, moist winds as the approach of rains, while the drier lowlands of Tharaka and Mbeere read strong, hot winds as a sign of dry conditions or drought. Increase in wind velocity in May and part of June indicates rains have subsided, and dusty storms in the afternoon in August mean the impending hot September weather when heat strikes like a mallet.

*“Do not wait [community members] for the winds (kiuo) to bring firewood to your home... tell them to cut loose tree branches near the homestead before kiuo season”*

*“Whirlwind, locally referred to as devils of women (ngoma cia aka)- the onset of fair weather that allows for weeding as the crop matures.*

We deduced that the onset of the rainy season is often signaled by faint lightning, observed at night in distant hills, usually to the east. This lightning is regarded by the community as a sign that rain is approaching, as it is commonly associated with the direction from which it rains.

*“There is lightening over Mbeu hills, gather your seeds ready for planting.... or we need to expedite land preparation... the rains are near” “Lightening has appeared once, twice thrice, reminding the herdsmen to repair their cattle sheds in Kiborione. i.e., the higher open ground where herdsmen take animals during the wet season.*

It is worth noting that communities around Mount Kenya interpret lightning as a positive sign to prepare for impending rains and seasonal changes. But in some African cultures, lightning is often viewed negatively and sometimes associated with witchcraft or a divine punishment [46]. In parts of Meru region boarding Mount Kenya forest, June-July period is characterized by chilly and foggy conditions, with communities interpreting low-lying clouds and light drizzle as onset of seasonal of elephant migration from the lower grasslands into the forest in search of food and water during the dry season. The onset of drizzling fog prompts households to harvest and remove crops from fields situated along the migratory routes to prevent damage.

*“Drizzles, and low hanging clouds remind the farmers to remove their maize from the farms to avoid attracting the elephants into the farms. We also caution herdsmen to avoid forested areas due to the risk of migrating elephants.*

**(iii) Celestial weather indicators**

Observations of the sun, moon, and stars were reported as central to forecasting seasonal changes. The crescent moon was reported as a marker of shifts in weather conditions, influencing decisions on planting and harvesting schedules. Additionally, the rainbow is a signal of coming rainfall. Some of the respondents said that the rainbow distributed rain across different localities to prevent destructive concentration in a single area. The presentation of moon and stars provided specific meanings to the weather changes.

*“Moon crescent facing upwards (Ruvia rwa Mbeere) meant plenty of rain…When the moon is surrounded by a halo known as Itiri (meaning “threshing floor*”). It signifies good weather and a bumper harvest, prompting farmers to plant larger areas of land. Another significant star observed during the April rains is Nkunku- e- Malima*, (the star for weeding season) “a bright star seen in the east signaling that it is time for farmers to intensify their weeding activities before the end of the rains to conserve moisture”.*

The star represents an early warning system that reinforces early planting and enhances resilience to fluctuating rainfall patterns. *“We can say that the moon, the sun, and the stars all serve to unite to provide communities with the information and preparedness they need to plan for their livelihoods”* These *celestial* bodies play a central role in local weather prediction, with the sun, moon, stars, and rainbows serving as dependable indicators of weather changes. The crescent moon’s orientation (Ruvia rwa Mbeere) is believed to signal abundant rains, while a halo around the moon (Itiri) indicates favorable weather and a good harvest. “*When the new moon hides behind a thin cloud, the farmers ready the hoes.”“If the sun rises dim through mist, expect the earth to drink early; sow your seed soon.”“When the stars tremble and the night wind turn east, the rains will follow the hills.”*These findings underscore the depth of community knowledge and interpretative skills in reading celestial rhythms and translating them into practical strategies for sustaining livelihoods. This knowledge is communicated through proverbs and sayings that keep the community focused on activities that are central to their survival.

**(iv) Somatic weather indicators**

The respondents explained that weather changes manifest as joint aches, headaches, fatigue, night sweets, nose bleeding, mostly associated with elderly people are indicators of weather changes. In addition, increased thirst and lethargy were frequently reported as signs that rains are imminent, with thirst understood as the body’s instinctual preparation for moisture and fatigue reflecting shifts in the surrounding atmosphere. Additional indicators such as night sweats, headaches, and generalized malaise were commonly associated with the approach of rainfall.

Furthermore, the occurrence of very hot, sunny, and dusty days accompanied by sweaty nights was consistently described as characteristic of the late September to early October period, preceding the onset of the short rains.

*“This period of unrelenting heat, (late September early October) is called thano Mukungugu, meaning the hottest season when the sun knocks like a Mallet” causing headaches, joint pains, and general body weakness.*

*“Normally, I don’t take plain water but if I do, because of extreme thirst, it must rain that day” The last signal for the impending rain is the change in the blowing of a cool refreshing afternoon breeze a few days before the rains, which brings an end to the relentless heat and gives farmers time to prepare their land and keep seeds ready.*

Findings from the Eastern Mount Kenya region show that indigenous weather indicators are interconnected. For example, the flowering of trees and pollen allergic reaction among some community members are a stronger confirmation that seasonal change is underway. In another case, the restless behavior of livestock, cloud formation or wind shifts, reinforces the expectation of rainfall. These connections highlight how different domains of observation plants, animals, and atmosphere are woven together into a coherent forecasting system.

*“When the trees flower and our noses itch, we know the rains are close…. “If the cattle grow restless and the wind turns, the clouds will not delay… “The dove sings, the sky darkens, and the earth prepares for rain.”*

**(v) Accuracy and reliability**

There are mixed perceptions regarding the accuracy. Some community members said that indigenous weather methods are no longer as reliable because of several factors including changing weather patterns destruction of environment through human activities such as charcoal burning which has killed all the indigenous trees that form part of indicators,

*When we were young, this neighborhood had a nice tree cover of yellow back Acacia (Mirera) which use to flower very nicely putting a nice scent into the air just before the rains, now those trees are gone..turned into charcoal.*

Community members generally felt that indigenous weather forecasting has become less accurate in modern times due to global changes that disrupt established patterns. While they acknowledged the value of traditional indicators, many observed that extreme events now exceed the scope of indigenous knowledge. Referring to the El Niño season, one elder from Meru explained,

“*We knew from our cycles that El Niño (mafuriko) would bring heavy rains, but the amount and destruction we saw was far beyond what our signs had told us.”*

At the same time, others emphasized that indigenous forecasting remains valuable because it adapts to local contexts. They noted that while global changes may alter the scale of events, local signs still provide guidance for day-to-day decisions such as planting, herding, and water use. As one farmer in Mbeere remarked,

“*Even if the big rains are stronger than before, our trees, winds, and animals still tell us when to begin preparing.”*

Together, these perspectives illustrate both the limitations and enduring relevance of indigenous forecasting under changing climatic conditions. They said that even the known indicators such as animal behavior and plant flowering no longer align consistently with rainfall patterns.

*“Sometimes it rains early, sometime there is too much heat in the middle of dry season, followed by a heavy downpour which leaves the farmers unsure if to start planting or to wait for the known season.*

Another community voice quipped, “*These days the signs sometimes deceive us; the trees flower but the rains may not come.*”

Others, however, emphasized that indigenous forecasts remain more dependable because they are localized and grounded in familiar environmental cues. A farmer from Mbeere explained,

***“*** *The radio tells us about the whole county, but our birds and winds tell us what will happen here in our village.”*

The methods have become less reliable under changing climate conditions, community’s awareness on the increasing unreliability of modern and indigenous methods leads them to consider both indigenous and scientific. They said that the government (meteorology) weather announcements can prompt a closer scrutiny of the local weather patterns.

*When we hear, about weather reports in the highlands east of rift valley, west of rift valley, central highlands, we start looking for signals that bring rain to our region to start preparing accordingly.*

These methods are considered reliable because they are rooted in close observation of small geographical areas and depend on well-established seasonal patterns. The increasing impacts of climate variability and the perceived inaccuracies of conventional meteorological forecasts have reinforced the community’s reliance on indigenous approaches, which are increasingly viewed as practical alternatives for anticipating weather under changing climatic conditions. A common joke about inaccuracy of modern meteorology says:

*“In Kenya, a common joke holds that when the weatherman predicts sunshine, people hang their clothes outside only for the rains to drench them moments later].”*

**(vi) Knowledge transmission and social authority**

The elders are the primary custodians of indigenous knowledge and interpretations skills of the weather. They play an important role in disseminating early information about environmental changes and extreme weather events. Their deep understanding of natural patterns and their lived experiences provide invaluable and reliable guidance. Elders act on behalf of communities. Their knowledge and advice inform decisions related to livelihood of community activities and strategies to mitigate the impacts of bad weather. Most importantly they hold the knowledge not as individuals but in trust as a community resource

*This knowledge is not mine; it belongs to the community. Mine is to report what the community has experiences over many, many years. That is why elders are always care to say, attribute everything to the community.*

*Those who possess these interpretive skills carry a responsibility to serve the community not themselves.*

They also command respect and undisputed status within their communities due to their knowledge and skills in safeguarding the community welfare. This role elevates their position and ensures that they are regarded with dignity. The physical limitations and care needs that often accompany old age managed through accommodation and tolerance which reinforces their significance in the cultural and environmental fabric of society.

*“It is good to allow elderly people to sleep like a well-fed child because, in their sleep, they listen to the winds interrogate the stars and feel with their bodies... they remind the community when to start preparing for impending weather changes.*

**Fig 4:**
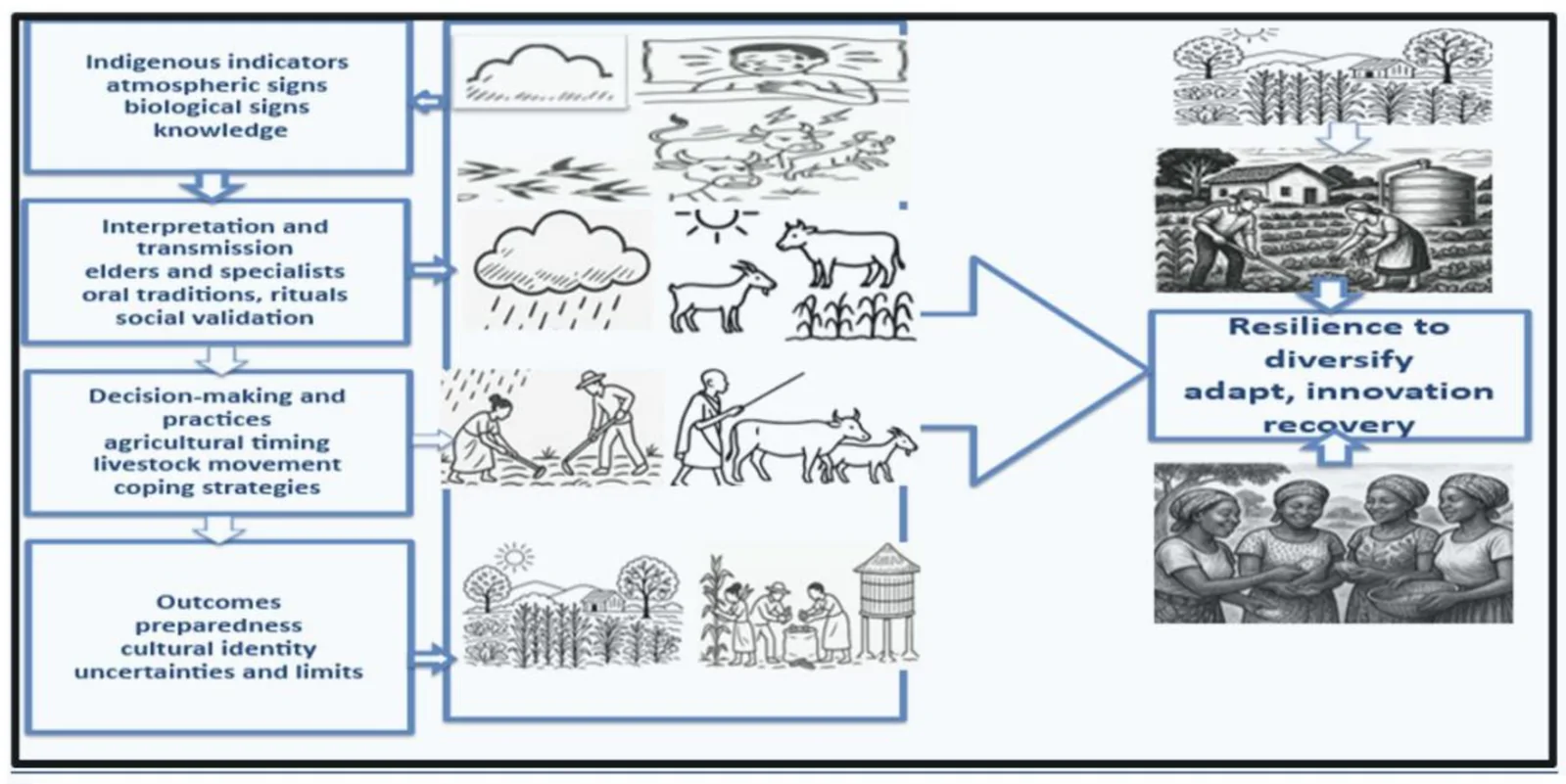
A flowchart of indigenous weather indicators, interpretation and contribution to community preparedness and resilience.

We also established that communities perceive these methods more localized and therefore relatively accurate and reliable but also subject to changing weather patterns. Despite the community trust in such methods there is a growing concern of lack of continuity in weather interpretation skills among the young generations a fact that threatens the continuity of such practices being of benefit to the communities. Indigenous weather indicators are interpreted by elders to inform livelihood decisions leading to preparedness of weather uncertainties. Fig 4 shows the overall outcome is adaptation, recovery and resilience.

## Discussion of findings

This study documented indigenous weather forecasting practices methods among communities in Eastern Mount Kenya region, an economically unique region dependent on rainfed cash crop and subsistence farming. These two sectors are vulnerable to current unpredictable weather patterns due to climate change [47,48]. Indigenous weather forecasting methods in the wake of climate change have been documented among various communities [49,] However, Eastern Kenya communities remain underrepresented, particularly with respect to indigenous weather forecasting practices that guide local decision-making and adaptation. [50,51]. Furthermore, a strong body of qualitative research that fully capturing the social and cultural factors that shape community vulnerability and resilience is needed, and that is the focus of this study contributes [52,53].

The onset of rains emerges as the most important seasonal event, around which nearly all predictions are centered. Across the four categories of indicators identified, the majority including the flowering of trees, the position of the moon, the appearance and calls of birds, and even bodily sensations are directly linked to anticipating rainfall. This finding underscores not only the practical importance of rainfall for livelihoods but also the deep cultural significance and enduring connection between communities and their natural environment. Similar emphasis on rainfall onset has been documented in other African contexts; for example, pastoralists in the Sahel closely observe plant phenology and animal behavior to anticipate the start of rains, which determines grazing movements [54]. Likewise, farming communities in Ethiopia rely heavily on environmental cues such as insect emergence and tree flowering to forecast rainfall onset, which guides critical decisions on planting and resource allocation [55]

The results of our analysis the indigenous weather forecasting methods used by the communities in eastern Mount Kenya, the mixed perceptions about their accuracy, the knowledge transmission, and social institutions and authority and how that knowledge is building on resilience and measures to mitigate the impact of climate change. These findings are supported by other studies which demonstrate similar outcomes [56]

Indigenous weather methods reflect a relationship with the environment that is rooted not in abstract data but in lived experiences accumulated over generations. For example, *“when the mututu bird calls repeatedly in the evening, we know for sure the rains are near*.” Such indicators are noteworthy not only for their proximity to the actual event but also for how their interpretation is woven into everyday life. These findings are supported by other studies, [57,58] which has established similar observations among pastoralist communities who interpret the behavior of certain birds, the flowering of specific trees, and the movement of livestock as biological and environmental cues for the onset of rains. Worth noting is other communities use the calling patterns of frogs, the timing of insect emergence, and changes in wind direction to forecast rainfall, embedding these interpretations within their cultural rituals and communal decision-making [59].

A significant finding was the community’s growing awareness that indigenous indicators are becoming less reliable due to disruption of weather patterns because of climate change. As one farmer observed, *“the signs we used to depend on, like the early calls of certain birds or the flowering of trees, no longer align with the rains as they once did*.” This observation is echoed by the findings among pastoral communities in northern Kenya and southern Ethiopia, who reported a declining trust in traditional indicators as rainfall patterns became increasingly variable. This report shows that the confidence in interpreting environmental signals such as animal behavior and plant changes is declining, and more caution is taken because the indicators no longer consistently align with actual weather outcomes [60].

This development brings about a need for adaption of policy development and institutional mechanisms to support such hybrid approaches. This view is supported by analytical evidence from the broader which confirms that the integration of indigenous knowledge with scientific forecasts increases trust, usability, and uptake of weather information among farming and pastoralist communities [61].

Our finding that the role of elders in weather forecasting is cherished by the community aligns with other studies that highlight the central role of elderly people in sustaining indigenous knowledge systems. Their insights knowledge and skills in interpreting the reinforces social cohesion, providing elders with a valued status. For instance, research among the Nganyi rainmakers of western Kenya emphasizes how elders accumulated knowledge anchors community trust in weather prediction, while studies in southern Ethiopia similarly show that the authority of elderly forecasters remains integral to community adaptation strategies [62].

In contrast, other studies show that when elders are not assigned meaningful social roles, they may be perceived as a burden to families and communities. For example, in some parts of sub-Saharan Africa, elderly persons, particularly those without land or productive capacity, are sometimes marginalized or may be accused of practices that hurt community’s member including witchcraft, resulting in neglect or mistreatment [63] Similarly, studies in urbanizing communities note that the absence of clear cultural roles for older people often results in their social isolation and diminished respect [64] These accounts highlight how the accommodation and esteem granted to elders in our study community, because of their central role in weather forecasting, contrast to societies where elders are viewed as a bother rather than valued custodians of knowledge.

A striking finding from our research is the strong emphasis placed on somatic or bodily sensations as indicators of impending weather change. Respondents frequently cited unusual headaches, joint pains, and sudden fatigue as reliable signs of imminent rainfall. While similar practices of somatic forecasting have been documented among elders in Tanzania’s Kilimanjaro region [65] the prominence of these indicators in our study suggests an especially intimate bodily–environment relationship. Comparative studies from West Africa also note that farmers in Burkina Faso and Ghana sometimes attribute bodily discomforts, such as dizziness or joint stiffness, to shifts in atmospheric conditions preceding rainfall [57]. Likewise, research in southern Ethiopia highlights that older pastoralists often interpret recurring body aches or mood swings as signals of approaching seasonal transitions [66]. Taken together, these findings point to a broader African pattern in which the human body is perceived not merely as a passive observer of environmental change but as an active sensor, deeply integrated into systems of local weather knowledge [58].

Regarding reliability and challenges this research revealed that the cherished role of indigenous weather indicators and trust due to consistency and geographical proximity of reliable results, there is a growing concern over changing weather patterns which disrupts the consistency of know signals. For examples, when rains come earlier than usual, they find farmers not prepared, when they delay, they cause inconsistent growth and sometimes loss of harvest and when they persist into the drying season, they lead to rotting especially of cereals. Communities’ member also linked the change in known weather indicators patterns to human activities especially the cutting of trees for charcoal burning. *“Most of the trees that used to bloom as signals before the rain is gone, cut down by us. We cannot read signs that are no longer there.”* This perception reflects wider discussions on how climate stress is eroding ecological knowledge. The issue is not that indigenous indicators are outdated, but that they depend on ecosystems whose rhythms have been disrupted, making some signs less reliable [67].

Integration with scientific forecasting an important insight emerging from the study, arising from the community’s call for complementarity between indigenous and scientific forecasts. While participants valued their own methods, many also appreciated advisories from the Kenya Meteorological Department, particularly when delivered through radio and television. However, there concerns over the applicability and most urgent needs: *“The government tells us about climate in future, El Niño and other information that we don’t really find useful. What we want to know is if it will rain in our village next week. That is why we still trust our birds and stars.”* This illustrates that while both systems are recognized, the community’s priority lies in forecasts that address immediate agricultural decisions and livelihood outcomes**, s**uch as when to plant or protect crops. It would help in advocacy for environmental protection through tree planting and good agriculture practice.

A key question arising from the findings was how this knowledge and skills of indigenous weather forecasting are being passed to the younger generations and their availability to support hybrid system of weather forecasting. Loss in this knowledge and skills was a recurrent theme among the elders we spoke to, who felt despite the urgent need to optimize agriculture productivity to cater for the needs of growing population, indigenous weather forecasting methods knowledge and skills are getting lost due to intergenerational gap. *“the younger generation do not look at the skies or listen to the winds or observe the bird migration to know the best time to prepare land for planting.* This intergenerational gap underscores the urgency of documenting and revitalizing indigenous knowledge systems before they are irreversibly lost [68].

This study advances ethno-meteorological research by identifying somatic or bodily sensations as a systematic forecasting domain, showing that the human body functions as an embodied environmental sensor within a coherent bio-cultural knowledge system. The findings further reveal a pronounced scale mismatch between national scientific forecasts and local decision-making needs, providing empirical support for hybrid, community-level forecasting models. The study also conceptualizes elders as informal climate governance institutions whose interpretive authority organizes collective adaptation. Finally, it introduces the concept of *ecological readability loss* the diminishing legibility of environmental cues due to degradation and demonstrates that intergenerational knowledge erosion constitutes a structural vulnerability that undermines adaptive capacity.

## Conclusion

This study demonstrates that indigenous weather forecasting in the Eastern Mount Kenya region operates as a dynamic, socially embedded, and ecologically grounded knowledge system. By documenting atmospheric, biological, celestial, and somatic indicators, the research shows that local forecasting practices remain central to everyday agricultural and livelihood decisions, even in contexts where formal meteorological information is available.

The findings indicate that climate change and environmental degradation are undermining the ecological cues that sustain indigenous forecasting, reducing the reliability of traditional indicators. This does not reflect a failure of indigenous knowledge but rather the disruption of the ecosystems on which such knowledge depends. In parallel, the study identifies a persistent mismatch between the scale of scientific forecasts and the localized, short-term information needs of rural communities.

The results provide evidence supporting the development of locally responsive, hybrid climate services that integrate indigenous and scientific knowledge systems. The study also highlights the critical role of elders as informal climate governance actors and identifies intergenerational knowledge loss as an emerging element of climate vulnerability.

Overall, the findings underscore the urgency of systematic documentation, preservation, and policy support for indigenous forecasting practices as part of broader strategies to strengthen adaptive capacity and sustain culturally grounded climate resilience.

The findings have several implications. First, they underline the potential of indigenous knowledge as a complementary resource for climate adaptation strategies at county and national levels. Integrating local forecasting indicators into extension services could improve the relevance of advisories for smallholder farmers. Second, preservation of this knowledge contributes to the cultural continuity of mountain communities, ensuring that forecasting is recognized not only as a technical practice but also as part of intangible heritage. Third, environmental conservation emerges as a key priority; the loss of indicator species and trees undermines both ecological balance and knowledge systems that depend on them.

### Research limitations

This study has several limitations that should be considered when interpreting its findings. First, the research relied primarily on qualitative methods and purposive sampling, which, while appropriate for capturing depth of indigenous knowledge, limit the generalizability of the results beyond the Eastern Mount Kenya region. The study draws on self-reported perceptions of forecasting accuracy, which may be influenced by recall bias and cultural expectations. In addition, although the study documents a wide range of indigenous indicators, it does not quantitatively assess their meteorological accuracy against instrumental weather data. Intergenerational dynamics were examined through participant narratives rather than longitudinal observation, which constrains the ability to assess temporal changes in knowledge transmission. Future research could strengthen this evidence base through mixed methods designs, longitudinal studies, and comparative validation with meteorological datasets.

### Future research directions

Future research should build on these findings through mixed-methods and longitudinal designs that systematically assess the predictive accuracy of indigenous indicators against instrumental meteorological data across multiple seasons. Comparative studies across agro-ecological zones would enhance understanding of how ecological change reshapes the reliability of ethno-meteorological knowledge systems. Further work is needed to examine gendered and youth perspectives to better understand intergenerational transmission pathways and barriers to knowledge continuity. Experimental co-production models that integrate indigenous forecasting with downscaled, hyper-local scientific climate services could provide actionable frameworks for hybrid climate information systems. Such research would strengthen the empirical and practical foundations for culturally grounded, locally responsive climate adaptation strategies. Further areas of research could focus on quantitative economic evaluation, funding mechanisms, youth engagement in knowledge transmission, and financial instrument innovation, including micro-subsidies, climate insurance, and income-generating opportunities linked to indigenous knowledge.

## Supporting information

S1 Data Anonymized Dataset. (XLSX)

S1 Appendix Appendix 1 COREQ. (PDF)

S2 Appendix Appendix II Consent form. (PDF)

S3 Appendix Appendix 111 Information Sheet. (PDF)
