# Supplementary material for: “When the Emerald wood dove calls, the earth prepares to drink”: Community elders lived experiences of indigenous weather forecasting in eastern Mount Kenya region
Source: PLoS One. 2026 Sep 8;21(9):e0355610. doi: 10.1371/journal.pone.0355610 (PMC13552957; doi:10.1371/journal.pone.0355610)
Supplement: Supplementary file 1 [file pone.0355610.s002.pdf]

Appendix 1: Consolidated Criteria for Reporting Qualitative Studies (COREQ): 32-Item Checklist

No. Item	Guide questions/description	Report on Page #
**Domain 1: Research team and reflexivity**		
Personal Characteristics		
1. Interviewer/facilitator	Which author/s conducted the interview or focus group?<br>Beatrice Warue and Kinyua Mucheke	Methods<br>(BW, KM,)
2. Credentials	Both PhD holders	BW: PhD in Business Administration & Management<br>KM PhD in Public Health
3. Occupation	What was their occupation at the time of the study? BW lecturer<br>KM Researcher/ Programs Advisor	BW Professor KM: Programs Technical Advisor
4. Gender	Was the researcher male or female?	BW: Female<br>KM: Male
5. Experience and training	What experience or training did the researcher have?	BW: Over 15 years of involvement in research<br>KM: Over 15 years of involvement in project design/ research
Relationship with participants		
6. Relationship established	Was a relationship established before the study commencement?	Yes/worked together
7. Participant knowledge of the interviewer	What did the participants know about the researcher? e.g. personal goals, reasons for doing the research	None
8. Interviewer characteristics	What characteristics were reported about the interviewer/facilitator? e.g. Bias, assumptions, reasons, and interests in the research topic	None
**Domain 2: study design**		
Theoretical framework		
9. Methodological orientation and Theory	What methodological orientation was stated to underpin the study? e.g. grounded theory, discourse analysis, ethnography, phenomenology, content analysis	Ethnography, Phenomenology,
Participant selection		
10. Sampling	How were participants selected? e.g. purposive, convenience, consecutive, snowball	Methods (purposive)
11. Method of approach	How were participants approached? e.g. face-to-face, telephone, mail, email	Methods. face to face

12. Sample size	How many participants were in the study?	Methods (81)
13. Non-participation	How many people refused to participate or dropped out? Reasons?	None
Setting		
14. Setting of data collection	Where was the data collected? e.g. home, clinic, workplace	Methods/home (Meru and Embu counties, Kenya)
15. Presence of non-participants	Was anyone else present besides the participants and researchers?	Methods (None)
16. Description of sample	What are the important characteristics of the sample? e.g. demographic data, date	Methods (Elderly men and women over 65 years of age who were permanent residents of the research areas)
Data collection		
17. Interview guide	Were questions, prompts, and guides provided by the authors? Was it pilot-tested?	Methods Yes, a Questionnaire was used and was pretested.
18. Repeat interviews	Were repeat interviews carried out? If yes, how many?	None
19. Audio/visual recording	Did the research use audio or visual recording to collect the data?	None (Methods)
20. Field notes	Were field notes made during and/or after the interview or focus group?	(Yes) Methods
21. Duration	What was the duration of the interviews or focus groups?	
22. Data saturation	Was data saturation discussed?	Methods
23. Transcripts returned	Were transcripts returned to participants for comment and/or correction?	Yes
**Domain 3: analysis and findings**		
Data analysis		
24. Number of data coders	How many data coders coded the data?	Methods (BW, KM)
25. Description of the coding tree	Did the authors describe the coding tree?	Thematic analysis
26. Derivation of themes	Were themes identified in advance or derived from the data?	Methods (Derived from the data)
27. Software	What software, if applicable, was used to manage the data?	N/A
28. Participant checking	Did participants provide feedback on the	Yes

	findings?	
Reporting		
29. Quotations presented	Were participant quotations presented to illustrate the themes/findings? Was each quotation identified? e.g. participant number	Yes: Results
30. Data and findings consistent	Was there consistency between the data presented and the findings?	Yes: Findings and discussions
31. Clarity of major themes	Were major themes presented in the findings?	Yes: Results
32. Clarity of minor themes	Is there a description of diverse cases or a discussion of minor themes?	Yes: Results
