# Supplementary material for: “When the Emerald wood dove calls, the earth prepares to drink”: Community elders lived experiences of indigenous weather forecasting in eastern Mount Kenya region
Source: PLoS One. 2026 Sep 8;21(9):e0355610. doi: 10.1371/journal.pone.0355610 (PMC13552957; doi:10.1371/journal.pone.0355610)
Supplement: Supplementary file 2 [file pone.0355610.s003.pdf]

Documentation of indigenous Methods of Weather Focasting in Eastern Mount Kenya Region.

Participant Consent to take part in research

I ………………………………………...................... voluntarily agree to participate in this research study.

I understand that even if I agree to participate now, I can withdraw at any time or refuse to answer any question without any consequences of any kind.

1. I understand that I can withdraw permission to use data from my interview within two weeks after the interview, in which case the material will be deleted.
2. I have had the purpose and nature of the study explained to me in detail and I have had the opportunity to ask questions about the study.
3. I understand that participation involves me sharing information on weather patterns in this region and the different methods used by the indigenous people to determine changes in weather and seasons.
4. I understand that I will not benefit directly from participating in this research.
5. I agree to my interview being audio-recorded.
6. I understand that all information I provide for this study will be treated confidentially and only for the purposes of the study.
7. I understand that in any report on the results of this research my identity will remain anonymous. This will be done by changing my name and disguising any details of my interview which may reveal my identity or the identity of people I speak about.
8. I understand that disguised extracts from my interview may be quoted in a conference presentation and journal publication.
9. I understand that signed consent forms and original audio recordings will be retained in secure place for a year
10. I understand that I am free to contact any of the people involved in the research to seek further clarification and information.

Sign: -- ----------------: Date……………………………

Signature of participant

I believe the participant is giving informed consent to participate in this study

Researcher name: ---------------------------Sign--------------------Date……………………
