# Supplementary material for: “When the Emerald wood dove calls, the earth prepares to drink”: Community elders lived experiences of indigenous weather forecasting in eastern Mount Kenya region
Source: PLoS One. 2026 Sep 8;21(9):e0355610. doi: 10.1371/journal.pone.0355610 (PMC13552957; doi:10.1371/journal.pone.0355610)
Supplement: Supplementary file 3 [file pone.0355610.s004.pdf]

Appendix 111: Information Sheet-Local Language Translated to English

Study Title/ ***Kiongo kia uvoro/kiongo kia jaria jakwariirua:*** **"When the Emerald Wood Dove Calls, the Earth Prepares to Drink": Community Elders Lived Experiences of Indigenous Weather Forecasting in Eastern Mount Kenya Region.**

/Giututu kiagamba, thi igagkunda: Aturi kuvesana kiririra kiao kia unduire wa imera/Kimeru Mututu jwagamba, nthi ikaithuraranira kunywa ruuji/ mantu ja withire jaria akulu ba njuri bategaga kumenya mugaruko jwa bingo nteerene cia kirimara kia gaiti.

Beatrice Warue (Principal Investigator) / ***(Munene wa uthuthuria/mutongeria wa uandiki bwa nteto iji) Stephen*** **Kinyua Mucheke** (Co-Principal Investigator / ***(Munini wa uthuthuria)mutetheria wa muandiki munene*** **Adelaide Lusambili** (Co-Principal Investigator/ ***unini wa uthuthuria) mutetheria wa muandiki munene***

Information Sheet ***/ Karatathi ko uvoro/ mantu jaria jakwarirua***

For participants over 65 years ***/ Avesani uvoro a kuma miaka 65/ KImeru: Aejani ba nteto, kuma miaka milongo ithanthatu gwita mbere***

Introduction / ***Kiambiriria/KImeru mwambirio***

We want to invite you to take part in a research study. This study is being conducted by Beatrice Warue and Kinyua Mucheke from Africa International University in Nairobi.

Participating in the study is voluntary without any coercion. Before you decide, you need to understand why the research is being done and what it will involve. Feel free to ask us if anything is unclear or if you want more information. We request you sign the consent form or give verbal consent, whichever you feel comfortable with only if you are sure you want to participate in the study. If you decide to join and change your mind later, that's okay! You are free to withdraw from the study at any time without giving a reason. **Kiembu** ***/Nitwakwamukira unyitanire natue guocania uvoro uyu utuletete guku kwenyu. Igwa wina wiyathi wa kunyitanira natue. Tumite university itagwa Africa International University. Kunyitanira natwe uvorori uyu nikwiendera gutari ne kucukumithua. Mbere ya utuve uvoro nwa tende werewe wocania wa uvoro uyu and gitumi kia guo. Igua win a wiyathi kuria kiuria o kinthe nigetha werewe wega.Wina wiyathi wa gwitikira kana urege gutuva uvoro uyu. Ukngitikira, tusainire karatathi gaka tugukuva kana uge na kanua gaku niwetikira. Ukiende gucijia meciria ati ndukwenda kuvesna uvoro, no wega.***

Kimeru***: Tugukwambiria ugwatanire natwi gututhetheria kuuthurania na kuandika mantu jaria jaturetete naja kwenu. Igua ucokeretue nkatho na kwithiria utina nkanja ugitutethia na jaria tukwenda kwaandika. Tumite Africa International University. Kwaraniria natwi mantu jaria tukwenda kumenya ni untu bwa ugue guciendela na gwitikiria gutina gukinyirua kinya aniini. Mbere ya twambiaria kwaraniria nteto iji ndenda wigua uciejene gwengwa na ujukie kanya kuuria uria wendekia kumenya untu bwa jaria jaturetete. Ukaigua utikuumba kana utikwenda kwaraniria natwi mantu jaja tukuria womba kuuga na tutiambirie, kana tugatigara aria tukethirwa tugukinya. Nitukuria usaini karatasi gaka, kwonania ugwitikirie gutucokeria biuria bikonie manju jaja. O kairi no uuge na mugambo jwaku ati ugwitikiria twaraniria o mbere tutarambiria.***

What is this study about / ***Ni uvoro uriku urocanua?/*****Kimeru:** ***Gitumi gia kuuthurania nteto iji***

Modern weather forecasting methods that rely heavily on meteorological data are becoming less unreliable due to the increasing unpredictability of weather patterns driven by climate change. Most agriculture-dependent communities and institutions are turning to indigenous weather forecasting methods to complement scientific approaches, enhance resilience, and make more locally relevant

decisions. There is a concern that the older people, who hold this knowledge are passing away and there is a risk of losing this vital information that contributes to the weather prediction. In this regard, the purpose of this research is to document the indigenous methods of weather forecasting in Eastern Mount Kenya Region . / ***maundu ma kumenya imera thiku ino mangasagwa ni thirikari na rimwe uria mauga tigwo gwikaraga. Aturi a guku ni arimi na nimatumagira kirira kia guku kwao kumenya imera an mavinda. And akuru aria maina kirira giki nimarathii na mbere na gukua , na tukaurwa ni kirira. Uvoro uyu turocania niwagututethia kwandika na kwiga kirira giki gitikaure and gigatethia aria magauka ivinda riria ri mbere yetu kumenya uria maithe metu mamenya mavinda na imera.***

***Kimeru**: Mantu jeegii bionaniria bia mugaruko jwa bingo bia mwaka birria bitumgaire umenyo bwa mauku ibiambiritie kuremwa gwitikika niuntu bwa kugaruka kwa riera ria nthi. Niunti bwa gitumi giki, athomi na kinya antu baria berigagira umenyo buu nikeenda bamenya uria bakaithuranire kiri urimi ibambiriitie gutumira ummenyo bwa gintwire kumenya na kwithuranira kurita ngugu cia uandi bwa bia kuria na uriithi bwa nyomoo. Ikwithiritwe kurina uguaa ati ummenyo bwa mugaruko jwa bingo bwa gintwire bugate bukithiraga niunti antu baria akuru barina umenyo bubu kabetite bakithiraga. Kwou kurina gitumi gia kuuthurania na kwaandika mantu jaja mbele ya igita riao kuuma nthi iji ritirathira.*

Why have you been invited? / ***Uthuritwe kuvesana uvoro uyu niki?/Kimeru: Gitumi gia gukuromba twaranirie***

You have been invited to take part in this study because of your experience and deep knowledge of the indigenous methods of weather forecasting based on gender, age, occupation and residency. in Region / ***Uthuritwe kuvesana uvoro uyu tondu wi na kirira kia mavinda na imera cia guku kwenyu, kurigana na miaka yaku, wira uria urutaga gwaku an iturari na gukorwa niuturite guku miaka mingi***

***Kimeru:** Gitumi gia gukuria twaranira niuntu uri mukari wa mwena juju na umwe wa baria barina uumenyo bwa mantu jamaingi ja migarukire ya bingo na kiria antu ba ruteere ruru bategaga kenda bamenya na kwithuranira mantu ja muturire.*

How will you use information collected in this study? */ **Mugatumira atia uvoro uria mugwocania? /**Kimeru**: Nteto iji igatumirwa atia?,***

The information you will give we will record it on question papers we have. This will help us put all information together for documentation. The records and information will be stored at Africa International University in a way that cannot be linked directly with your name. Please let us know if you have any concerns about this./ ***Nitukwandika uvoro uria ugutuva kwi maratathi maria turatumira kuria ciuria. Mandiko mau tugatumira kwandika rivoti ya kirira kia guku kwenyu. Uvoro ucio ukaigwa Africa International University na ritwa riaku ritikamenyithanwa utavesanite rutha.***

***Kimeru:** Tukandika jaria jonthe ugatwira na njira ya gucokia biuria na nyumene tuuthuranie macikio ja bothe baria tukaria nabo na twandike nteto cionthe amwe iri riboti imwe. Macokia jaku jatithirwa jarina riitwa riaku na jakaithwa obisine cia Africa International University. Jaja nijo mawatho jaria tuthingataga gukaria riitwa riaku wathiria kwaria natwi. Guti muntu umba gutwiiria na kumenya uria waugite mantune jaja tiga mwandiki wenka.*

What will happen to the results of the study? / ***Rivoti ya uvoro uyu igatumirwa atia?/***Kimeru*** Riboti na jaria jakaandikwa jagatumirwa atia***

The results of this study will be used in several ways to help others learn from our research. Once we've collected and analysed all the information, we'll bring community members together to present our findings and discuss practical ways preserving the indigenous weather forecasting approaches.

We will also share the results with policymakers, present the results at scientific conferences, and publish in scientific journals. / ***Nitugacoka kwi mwue ringi tumwire rivoti ya uvoro uria mutuvete na twitikanie uria tungivota kuiga kirira giki kia imera na mavida. Nituka vesana rivoti ino micemaniori and kwi aria matucaga mawatho ma gutumirwa kuringana na rivoti. Kimeru: Mantu jaria bugatwira kithomone giki jagatumirwa na njira mwanya mwanya kenda antu bangi bairitana najo. Twathiria kwandika tukeeja ukairi tubwee kanaya ga kuthikiira jaria bwatiwra kethira ni ja mma, na nyumene tukenenkari ripoti yetu kiri athomi nabaria baandikaga mibango ya gutethia mugongo mantu ja urimi na urithi bwa nyomoo.***

Who is funding the research? / ***Nuu uravesana mbeca cia kungania uvoro uyu?/Kimeru:Nuu uritite mbeca cia kuuthurania gwa nteto iji.***

The research is self-funded by BW and KM who are organisers of the project. /***Beatrice Warue na Kinyua Mucheke maravuthira mbeca cia kungania uvoro uyu/ Kimeru: Mbeca cia kuuthurania nteto iji ni cia aandiki baba bairi, Beatrice Warue na Kinyua Mucheke.***

Who has reviewed the study? / ***Nu uvesanite rutha rwa guocania uvoro?/ Kimeru. Nuu ucunkunite na uejanite ruutha rwa uthurania bwa nteto iji?.***

Research studies like this one are looked at by an independent group of people, called a Research Ethics Committee, whose main goal is to protect your interests. This study has been reviewed and approved by the Africa International University and NACOSTI/KENYA Research Ethics Committees, as well as the Meru and Embu County governments/ /***Ungania wa uvoro uyu niwitikiritue ni kamiti iria iroragia uma wa uvoro kuma Africa International university, , aria mavesanage rutha rwa guocania movoro metagwa NACOSTI na rutha kuma county cia Embu an Meru. Kimeru. Uthurania bwa nteto iji burungamagirwa ni rwangi rwa mantu ta jaja rwa Africa International University na ruteere rwa thirikari ni wobisi itagwa NACOSTI. Nkuruki ruutha rwa kuuthurania nteto jiji rwaejanirwe na obisi cia counti ya Embu na Meru.***

Who can I contact if I have a problem or a question? / Kimeru: Nu ungiaraniria nake ukienda kuria kiuria?/Nuu umba kwaraniria nawe ukethorwa uri na kiuria?

If you have a problem or question about any aspect of this study, you should ask to speak to the researchers who will do their best to answer your questions. The names and contact details of the principal researchers are below: ***/ Aria marocania uvoro uyu ni Beatrice na Mucheke aria marugamirire wocania wa uvoro. Maritwa mao na namba cio cia thimu ciandikitwe ava thi ya uvoro uyu. Kimeru: Aandiki ba nteto iji ni Beatrice na Mucheke nibo barungamirite mantu jaja. Maritwa nan amba cia thimu nicio iji nthi aja.***

Dr. Kinyua Mucheke	Telephone +25 4722446498
Co- Principal investigator	Email: skmucheke1930@gmail.com
Prof. Beatrice Warue	Telephone +25 4721607317
Principal Investigator	Email: warue.daea@gmail.com
